# Seismic evidence for flow in the hydrated mantle wedge of the Ryukyu subduction zone

**DOI:** 10.1038/srep29981

**Published:** 2016-07-20

**Authors:** Takayoshi Nagaya, Andrew M. Walker, James Wookey, Simon R. Wallis, Kazuhiko Ishii, J. -Michael Kendall

**Affiliations:** 1 Graduate School of Environmental Studies, Nagoya University, Nagoya 464-8601, Japan; 2 School of Earth Sciences, University of Bristol, Bristol BS8 1RJ, UK; 3 Graduate School of Environmental Studies, Tohoku University, Sendai 980-8579, Japan; 4 School of Earth and Environment, University of Leeds, Leeds LS2 9JT, UK; 5 Graduate School of Science, Osaka Prefecture University, Sakai 599-8531, Japan

## Abstract

It is widely accepted that water-rich serpentinite domains are commonly present in the mantle above shallow subducting slabs and play key roles in controlling the geochemical cycling and physical properties of subduction zones. Thermal and petrological models show the dominant serpentine mineral is antigorite. However, there is no good consensus on the amount, distribution and alignment of this mineral. Seismic velocities are commonly used to identify antigorite-rich domains, but antigorite is highly-anisotropic and depending on the seismic ray path, its properties can be very difficult to distinguish from non-hydrated olivine-rich mantle. Here, we utilize this anisotropy and show how an analysis of seismic anisotropy that incorporates measured ray path geometries in the Ryukyu arc can constrain the distribution, orientation and amount of antigorite. We find more than 54% of the wedge must consist of antigorite and the alignment must change from vertically aligned to parallel to the slab. This orientation change suggests convective flow in the hydrated forearc mantle. Shear wave splitting analysis in other subduction zones indicates large-scale serpentinization and forearc mantle convection are likely to be more widespread than generally recognized. The view that the forearc mantle of cold subduction zones is dry needs to be reassessed.

Antigorite-bearing serpentinite is a key agent for the recycling of H_2_O, carbon, sulfur and other elements from the surface environment into the Earth’s mantle^1^,^2^,^3^,^4^. Antigorite is also thought to play a key role in the development of episodic tremor and slip in subduction zones^5^,^6^,^7^ and its strong anisotropy is thought to be an important cause of seismic anisotropy in the mantle wedge of convergent margins^8^,^9^. Therefore, it plays a central role in the dynamics of convergent margins. However, the distribution and proportion of antigorite in the mantle wedge of convergent margins are not well known. Petrological studies suggest that the depth at which subducting slabs dehydrate is strongly controlled by the age of the slab: young warm slabs release most of the available fluid in the forearc region e.g.^10^,^11^. This implies that antigorite-bearing hydrated forearc mantle is much more completely developed in relatively warm subduction zones than in cold e.g.^6^,^8^,^10^,^12^,^13^. The results of our study in the Ryukyu arc present a challenge to this idea.

One of the most widely used ways to recognize the presence of antigorite in the mantle is through measuring seismic velocities. Experiments show that rocks consisting mainly of antigorite are associated with relatively low values of Vp and Vs (Vp = ~6.5–6.7 km/s, Vs = ~3.4–3.7 km/s), and with higher Vp/Vs ratios (Vp/Vs = ~1.8–1.9)^14^,^15^ than those of dry olivine-rich mantle (Vp = ~8.0–8.6 km/s, Vs = ~4.5–4.9 km/s and Vp/Vs = ~1.7–1.8)^14^,^16^. An important factor not usually taken into account in this type of analysis is the seismic anisotropy of antigorite: ranging from maximum values of ΔVp_max_ = 46% and ΔVs_max_ = 66% to almost zero for vibrations within the basal (001) plane^9^. For single crystals, the Vp/Vs ratio can vary from 1.2 to 3.4 (Vp = 5.6–8.9 km/s, Vs = 2.5–5.1 km/s) for different seismic wave propagation paths^9^. As summarized in Fig. 1, depending on the angle of incidence between the seismic ray path and the (001) plane of antigorite, the values of Vp/Vs, Vp and Vs may all be indistinguishable from values expected for mantle that lacks serpentine minerals^17^. Shear wave splitting measurements provide robust evidence of seismic anisotropy and can potentially be used to map out the presence of serpentine and its alignment in the mantle wedge.

In most seismic studies of the forearc mantle, it is assumed that antigorite grains are randomly oriented within the rock and anisotropy can be ignored. However, numerous recent studies of the crystal orientations of antigorite in serpentinite have shown that antigorite is rarely randomly oriented e.g.^18^,^19^,^20^,^21^. These results highlight the need to consider the anisotropy of antigorite when using seismic tomography to examine the degree of serpentinization of the mantle wedge. The simplifying assumption of an isotropic medium may be one of the main factors contributing to the great variation in antigorite contents estimated from the seismic velocities within a single subduction zone: 15–100% in Cascadia^13^,^14^,^22^,^23^,^24^, 20–99% in Kanto, central Japan^14^,^25^, 15–90% in Nicoya Peninsula, Costa Rica^14^,^15^,^26^, 15–77% in Kii peninsula, Japan^14^,^27^, 15–77% Western Shikoku, Japan^14^,^27^ and 30–100% in the Marianas^14^,^28^. Only a small part of this variation can be explained by other differences, such as the choice of database used for the seismic velocities of antigorite.

Some of the best seismic evidence for the presence of antigorite and its distribution in subduction zone mantle wedge comes from the Ryukyu convergent margin^8^ (Fig. 2). In this region the large delay times associated with S-wave splitting are much greater than those which might be produced by crustal cracks or olivine-rich mantle^8^,^29^ alone. This has led workers to propose the presence of a 10-kilometer scale thick antigorite-rich shear zone parallel to the subduction boundary^8^ with a foliation defined by strongly aligned grains of antigorite. The presence of this foliation imparts a strong anisotropy to the rock, which can help account for the observed large delay times^8^.

This attractively simple model has a significant flaw: large delay times are only predicted for intra-slab events that propagate along the shear zone before reaching the surface. Teleseismic events should propagate at high angles to the proposed shear zone and show very low degrees of anisotropy similar to non-serpentinized mantle^9^,^17^. The observed delay times are, in fact, similar for both intra-slab and teleseismic events^30^.

To account for this observation, several previous studies have proposed that antigorite domains with steeply dipping foliation may be present^9^,^17^,^21^,^29^. Important as they are, these studies either lack consideration of ray paths^9^,^17^,^21^ or do not incorporate the elastic constants for antigorite^29^. These limitations mean that the full potential of seismic anisotropy to assess the proportion and distribution of antigorite in the forearc wedge has not been developed. Here, we use a combination of information from the fields of both petrofabrics and seismic modeling to better understand the distribution of antigorite in the mantle wedge and test the viability of our results with geodynamic modeling of convergent margins.

## Results

### Modeling the seismic anisotropy of the Ryukyu arc

Here we present results from a new model that calculates the S-wave splitting caused by propagation through anisotropic domains where both the strength and orientation of the anisotropy can be independently assigned. We apply this model to the well-documented seismic events in the Ryukyu forearc and show how knowledge of the ray path geometry can be combined with S-wave splitting to constrain the structure of the shallow mantle and hence infer the distribution and proportion of antigorite. Our model divides the wedge mantle into a 2 km grid with each grid point assigned an anisotropy. We then calculate the S-wave splitting expected at the Earth’s surface for different events with different ray paths. To account for the large trench-parallel fast Vs associated with both teleseismic and intra slab events, we investigate the effects of incorporating several domains of foliated antigorite mantle with the orientation of the associated anisotropy ranging from slab-parallel to vertical.

**Figure 3:**
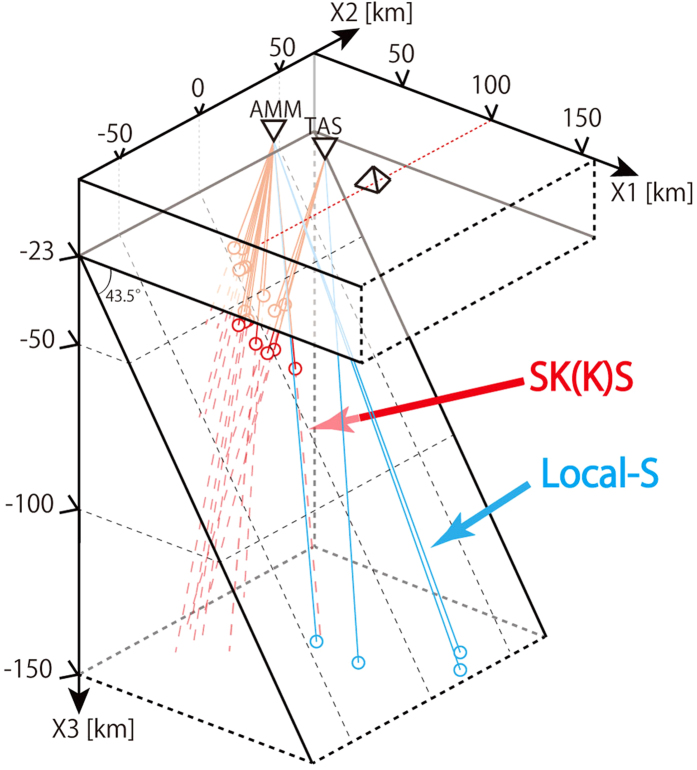
Diagram of ray paths used for calculation of S-wave splitting. Red and blue lines denote observed ray paths of teleseismic SK(K)S phases and local-S phases, respectively. The intersection of these paths with the subduction boundary is shown by circles of the appropriate color. The S-waves are recorded in two locations (AMM and TAS), which are indicated by the open inverted triangles. The triangular prism and the red dashed line denote the position of the volcanic arc. For ease of illustration, the locations of AMM and TAS have been projected along the X2-axis. The distances between seismic stations and the volcanic arc or the trench are not altered by this projection. The thickness of the continental crust is taken from ref. 29 and references therein and the locations of the seismic stations, ray paths and the distances between the volcanic arc and the seismic stations are taken from refs 29,30,32.

For our calculations, we use measured crystallographic preferred orientation (CPO) patterns of natural antigorite schist from the Happo area, Central Japan measured by an Electron Backscatter Diffraction (EBSD) system and reported in detail in ref. 31, and used this to derive the Voigt-Reuss-Hill average of the seismic elasticity of this antigorite schist using elastic constants of antigorite single crystals obtained from ref. 9. The effect of using other reported CPO patterns is also investigated. We calculated the S-wave splitting expected at locations corresponding to the seismic stations AMM and TAS in the Ryukyu Arc for several recorded events with known ray paths (Fig. 3)^29^,^30^,^32^. We then compared the model results with the actual observations^29^,^30^,^32^. To simplify this comparison we use the average values of the calculated and observed delay times, *dt*, and fast directions, *φ*, for the teleseismic SK(K)S phases, the local-S phases, and both phases together. To estimate the average values of *dt* and *φ*, we determined the best fitting curves to the expression: *Si* = *dt sin* (2(*θ*−*φ*))^33^, where *Si* is the splitting intensity and *θ* is the incoming polarization angle equal to the initial splitting angle for each event. This methodology is applied to each model and to all observations (see Supplementary Fig. S1 observed and model results for each path).

**Figure 4:**
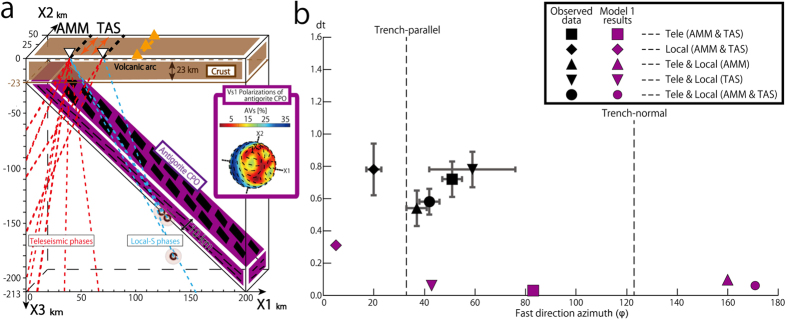
Model 1 of antigorite-bearing serpentinite and comparison between the observed results and S -wave splitting calculated using model 1 for the Ryukyu subduction. (**a**) *Model 1*. Strongly foliated antigorite domain (purple) parallel to the subduction boundary. We used 3-D ray paths as shown in Fig. 3, but for the purposes of illustration, seismic stations, seismic sources and tele- and local- seismic ray paths are projected onto the X1–X3 plane based on refs 29,30,32. These are denoted by open triangle, brown spheres and red and blue dotted lines, respectively. The orange arrows denote observed fast directions. The Vs_1_ polarizations figure (3-D distribution of the polarization direction of the fast S-wave passing through various directions) with color shading for AVs (the polarization anisotropy of S-waves owing to shear wave splitting, 200(Vs_1_ − Vs_2_)/(Vs_1_ + Vs_2_)^59^) was prepared using software that combined the two MATLAB toolkits, MTEX and MSAT and incorporated an antigorite CPO from ref. 31. (**b**) The relationship between averaged delay times (vertical axis) and fast direction azimuths (horizontal axis) for observed data in black with 95% CL in gray and model results in purple. Squares, rhombi and triangles denote the results for tele-, local- and combined seismic phases obtained from both AMM and TAS seismic stations, respectively. Inverted triangles and circles denote the results for combined seismic phases for each AMM and TAS seismic stations, respectively. This calculation includes an assumed crustal anisotropy of 0.3 s with trench-parallel splitting^35^,^36^.

**Figure 5:**
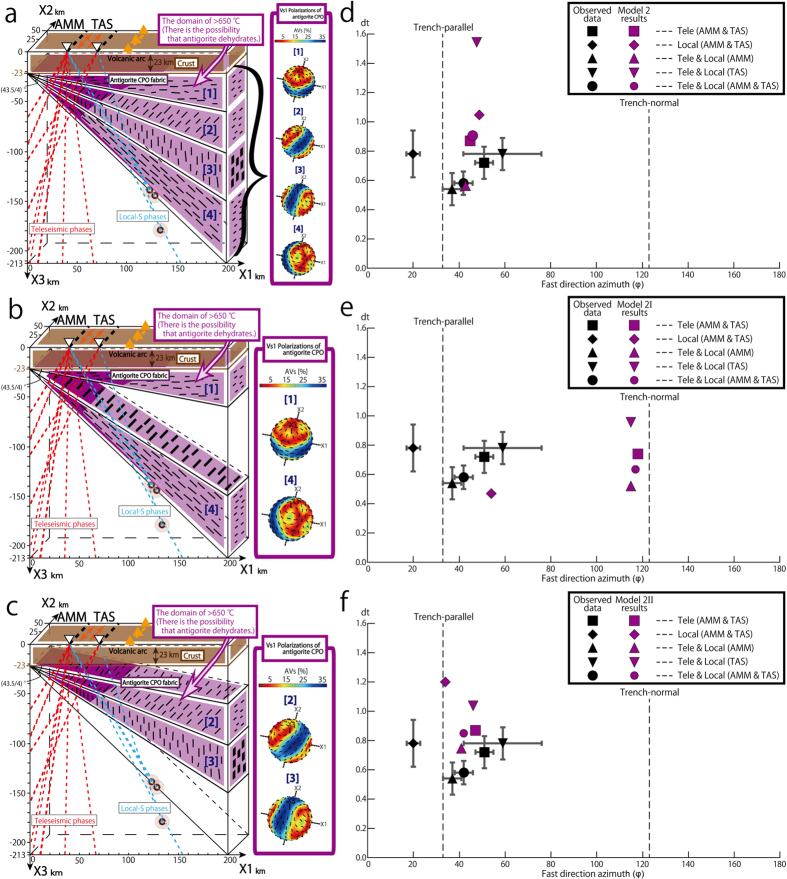
Model 2, 2I and 2II of antigorite-bearing serpentinite and comparison between the observed results and S-wave splitting calculated using model 2, 2I and 2II. (**a**) Model 2 antigorite distribution. The antigorite foliation changes from horizontal to parallel to the subducting slab. Light purple domains represent the areas where thermo-mechanical modeling^29^ suggests that temperatures will be too high for the stable existence of antigorite, but in this model antigorite presents throughout the modeled region. Color shading for AVs is shown on Vs_1_ Polarizations figure. All others features are shown in the same way as in Fig. 4a. (**b**) Model 2I antigorite distribution. The geometry in model 2I uses the two antigorite-bearing domains (areas [1] and [4]) used in (**a**). All others features are shown in the same way as in (**a**). (**c**) Model 2II antigorite distribution. The geometry in model 2II uses the two antigorite-bearing domains (areas [2] and [3]) used in (**a**). All others features are shown in the same way as in (**a**). (**d,e,f**) The relationship between averaged delay times (vertical axis) and fast direction azimuths (horizontal axis) for observed data in black with 95% CL in gray and model 2, 2I and 2II results in purple, respectively. This calculation includes an assumed crustal anisotropy of 0.3 s with trench-parallel splitting^35^,^36^. All others features are shown in the same way as in Fig. 4b.

**Figure 6:**
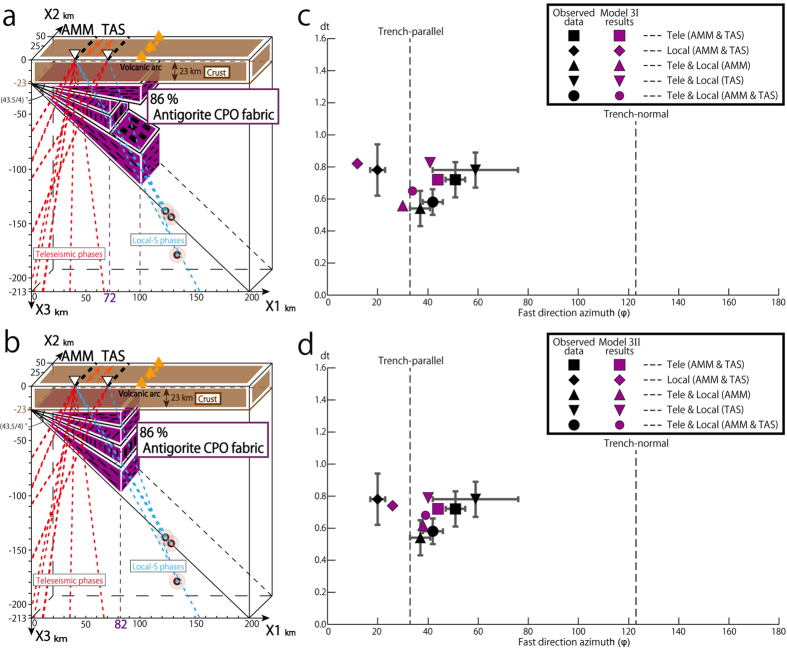
Model 3I and 3II of antigorite-bearing serpentinite and comparison between the observed results and S-wave splitting calculated using model 3I and 3II. (**a**) *Model 3I*. The vertically foliated antigorite domain extends to 72 km, and the horizontal and slab-parallel foliated antigorite domains extend to 100 km from the tip of the wedge mantle. All others features are shown in the same way as in Fig. 4a. (**b**) *Model 3II*. Horizontal extent of serpentinite-bearing domain is 82 km from the tip of the wedge mantle. All others features are shown in the same way as in (**a**). (**c,d**) The relationship between averaged delay times (vertical axis) and fast direction azimuths (horizontal axis) for observed data in black with 95% CL in gray and model 3I and 3II results in purple, respectively. This calculation includes an assumed crustal anisotropy of 0.3 s with trench-parallel splitting^35^,^36^. All others features are shown in the same way as in Fig. 4b.

In order to understand how the seismic anisotropy recorded in the Ryukyu subduction zone relates to structures within the mantle of this region, we examined six different models (Model 1, 2, 2I, 2II, 3I and 3II) all of which include domains of foliated antigorite serpentinite. The model calculations offer clear quantitative confirmation of the earlier suggestions that steeply dipping domains of foliated antigorite are required to account for the observed anisotropy in the Ryukyu Arc^9^,^17^,^21^,^29^ (Figs 4, 5, 6 and Supplementary Fig. S1). We are also able to examine how varying the proportion and distribution of antigorite-bearing domains in the mantle affects the results. Data for the teleseismic events that propagate the shallow forearc mantle where thermal calculations show that antigorite can stably exist^29^ gives an estimate for the percentage of serpentinite present in the Ryukyu forearc mantle arc. Next, we make use of the ray paths for local events that come from the back-arc side to estimate how far the domain of antigorite-bearing mantle extends towards the back-arc.

#### Model 1

The reference model, which we call model 1 shown in Fig. 4a follows previous suggestions for the structure of this region^8^ and has a single 10 km thick layer of antigorite parallel to the subducting slab. Antigorite in this model occurs as a zone parallel to the subduction boundary, it has a strong foliation defined by aligned antigorite. The average of the intra-slab local-S phases arriving at both seismic stations from the back-arc side of the margin show fast shear wave directions close to those observed, but the delay times are relatively small and do not closely match the observed values (Fig. 4b). In addition, the average of the teleseismic phases arriving mainly from the forearc side of the margin cannot adequately account for the observed delay times even if the thickness of the antigorite layer is changed (Fig. 4b). The calculated averages of both the teleseismic and local phases for each station show a much smaller delay time than the observations (Fig. 4b). Overall, results for this model are not consistent with the observations recorded at both seismic stations (Fig. 4b).

#### Model 2

In model 2 the antigorite foliation in the area beneath the continent crust is sub-horizontal (area [1] in Fig. 5a), and the foliation in the three remaining areas is set so that it changes by an angle of 45.5 degrees around the trench-axis (X2-axis in Fig. 5a) until the antigorite foliation is parallel to the subducting slab (areas [2]–[4] in Fig. 5a). This results in a domain of mantle with a vertical foliation corresponding to aligned (001) planes of antigorite. The presence of such a domain would imply the forearc mantle consists of domains where flow is not only parallel to the subduction boundary but also at a high angle to it. This type of spatial variation in flow direction implies the existence of large-scale mantle flow in the hydrated forearc mantle. We emphasize that this represents an intermediate stage in developing a more adequate model and the presence of antigorite throughout the modeled region includes areas where thermo-mechanical modeling of the Ryukyu arc region^29^ suggests temperatures will be too high for the stable existence of this mineral (Fig. 5a–c).

The results of calculations for model 2 show the seismic waves arriving from both the back- and fore-arc sides of AMM and TAS all show fast shear wave anisotropies sub-parallel to the trench. In addition, all delay times are greater than in model 1 and closer to the observed values. (Fig. 5d).

#### Models 2I and 2II

To examine in more detail the causes of the fast vibration direction sub-parallel to the trench and the large delay times, we separated the effects of the two antigorite-rich domains: one where the foliation is parallel either to the boundary between the continental crust and mantle or the subducting slab (model 2I as shown in Fig. 5b), and a second domain with a sub-vertical foliation (model 2II as shown Fig. 5c).

In agreement with the results of the calculations for model 1, the results for model 2I show the averaged local-S phases are associated with fast shear wave anisotropy sub-parallel to the trench (Fig. 5e). This means the antigorite domain parallel to the base of the continental crust doesn’t strongly affect the shear wave splitting. However the results for the averaged teleseismic phases and for a combination of teleseismic and local phases show the large delay times are associated with a trench-normal anisotropy (Fig. 5e). This indicates that the foliated antigorite domain parallel to the base of the continental crust causes trench-normal splitting of teleseismic phases. In addition, averages of both types of waves for each station are characterized by trench-normal anisotropies clearly showing that model 2I does not contribute to the results of model 2 that are close to the observations (Fig. 5e).

In contrast, the results of calculations for model 2II, show that seismic waves arriving both from the back- and fore-arc sides of AMM and TAS all show fast shear wave anisotropies that are subparallel to the trench and associated with large delay times (Fig. 5f).

These results show that the antigorite domains with a foliation (and hence (001) antigorite planes) parallel to the subduction boundary and a steep foliation are contributing to the trench-parallel fast directions and the large delay times for the local-S phases. In contrast, for the teleseismic phases, the antigorite domain with a steep foliation is the main cause of the trench-parallel fast directions and the large delay times.

#### Models 3I and 3II

In summary, models 1, 2, 2I and 2II show that for local-S phases the antigorite domains with foliations oriented either parallel to the subducting slab or subvertical are the main cause of the trench-parallel anisotropy and large delay times that are close to the observed values. For teleseismic phases, the domain with a sub-vertical antigorite foliation is the main cause of the trench-parallel anisotropy and large delay time and this domain is required to account for the observed results. In models 2, 2I and 2II antigorite-rich domains are distributed in thermally unrealistic areas. However, even when the thermal structure is taken into account, all ray paths pass through serpentinite-rich domains for at least part of their trajectory. The very strong anisotropy of antigorite means that ray paths sub-parallel to the foliation of antigorite should be associated with strong shear wave splitting even if only part of the ray path passes through a domain with a high proportion of foliated antigorite serpentinite. Analysis of the observed ray paths, their splitting times and relationships with the geometry of Ryukyu arc imply that the antigorite-rich domains extended at least ~60 km towards the back arc from the tip of the wedge mantle. In models 3I and 3II shown in Fig. 6a,b, respectively, we examined the results of changing the proportion and distribution of the antigorite domain in model 2 to optimize the fit between the observed and modeled results. These models show that a forearc domain consisting of about 86% antigorite shows a good fit with the observed properties of the teleseismic phases (Fig. 6c,d and Supplementary Fig. S2).

In model 3I the distribution of antigorite schist is considered to broadly follow the expected thermal profile in convergent margin where convection of the mantle wedge is induced by coupling between the downgoing slab and mantle wedge at depths greater than about 100 km along the slab interface—a depth which is directly below the volcanic arc in the Ryukyu arc (see Fig. 6a). In order to match the observed data, the antigorite area close to the crust and subducting slab must have a limited distribution equivalent to a horizontal distance of 100 km from the tip of the wedge mantle. This is in good agreement with predictions from thermal modeling^29^. Further analysis of this model provides an estimate of the minimum distribution of vertical foliation. We adopt the values for the proportion of antigorite derived from the analysis of teleseismic phases, and search for the distribution of antigorite within the wedge that best accounts for the observation of local-S phases. The size of the vertically foliated antigorite domain needs to be reduced to a distance of 72 km from the tip of the wedge mantle to obtain the best fit with the observed values (Fig. 6c). Next, we consider a model that shows the minimum distribution of slab-parallel foliation under the assumption that it shows the same lateral extent as the domain with a vertical foliation. In model 3II we assume antigorite is restricted to an 82 km wide domain in the forearc mantle (Fig. 6d). This model shows a good correspondence with the observed data. Overall both model 3I and 3II are consistent with the predicted thermal structure in the wedge mantle^29^ and also match the S-wave splitting results for both data sets.

#### Effect of uncertainties on the estimation of the proportion and distribution of antigorite

In this modeling we used the antigorite CPO measured by an EBSD system from natural antigorite schist in Central Japan^31^ as a reference material. However, we compare these results to the full range of antigorite CPO patterns reported in the literature to examine the effect of using other natural antigorite CPO patterns reported for subduction zone material, and then we include a review of published data and incorporate this range in our estimates. The reference antigorite CPO is characterized by a *b*-axis concentration parallel to the mineral lineation and a *c*-axis concentration normal to the foliation (B-type antigorite CPO); similar examples have been reported from many localities e.g.^18^,^19^,^20^,^21^,^31^,^34^. A distinct type of antigorite CPO has also been reported with the position of the *a*- and *b*-axis concentrations reversed (A-type antigorite CPO) e.g.^8^,^9^,^21^, which differs in that the crystallographic axis is parallel to the mineral lineation (the shear direction in the case of deformation experiments). Therefore we prepared the Vs_1_ polarizations figure with color shading for AVs using an antigorite CPO from ref. 31. As a result, each small segment on the Vs_1_ polarization figure (Figs 4 and 5) represents the trace of the polarization plane on the point at which S_1_ penetrates the sphere and the Vs_1_ polarization and AVs of antigorite CPO show similar characteristics irrespective of the orientation within the (001) basal plane. The *a*- and *b*-axes are oriented sub-parallel to the (001) plane, signifying that the seismic properties are very similar for both types of CPO and the choice of antigorite CPO has only a minor affect on the conclusions of this study. Antigorite CPO from ref. 31 used for this modeling shows the strongest AVs (<~37%) for all reported A- and B-type antigorite CPOs with the exception of ref. 9. The AVs of antigorite shows only a weak pressure dependence up to ~6 GPa (corresponding to a depth of ~200 km)^17^. Incorporating stronger CPO patterns in our calculations would reduce the amount of antigorite required to account for the observations. For instance, use of the very strong CPO reported in ref. 9, results in an estimate of antigorite content of 54% and we regard this as a minimum estimate. A review of the effects of using other available antigorite CPO patterns is presented in Supplementary Fig. S2. The estimate would be reduced by using data for a single crystal. However, this is an unreasonable approximation to natural samples and we do not take this approach. In estimating the minimum proportion of antigorite, we also include the range of possible values implicit in the various averaging schemes used to derive elastic anisotropy from CPO patterns. This is a usually ignored but potentially important source of uncertainty. The end-member cases are the Reuss (constant stress) and Voigt (constant strain) averages, which should cover the full range of possibilities for averaging schemes. The results for both these averaging schemes were included in our range of uncertainties. We consider the variability in natural CPO patterns of antigorite and different models (the Reuss and Voigt averaging schemes) for calculating anisotropy as uncertainties in the calculations shown in Supplementary Figs S2 and S3.

A detailed shear wave analysis in Taiwan shows a maximum value of 0.30 s for crustal anisotropy and this is associated with a trench-parallel fast direction^35^. This area is along strike of the Ryukyu arc and the analysed domain shows a very similar crustal thickness (~20 km) to the Ryukyu arc^35^. These observations are in good agreement with S-wave delay times derived from measurements of P-wave anisotropy (≤0.3 s for a crustal thickness similar to that seen in the Ryukyu arc)^36^ and the generally observed values for the crust in subduction zones with maximum delay time values of 0.2–0.4 s^37^,^38^. These values of crustal anisotropy are small compared to the delay times observed in the Ryukyu arc of 0.75–1.25 s^30^ but important when considering appropriate error estimates. In our modeling we use 0.3 s for the delay time as an upper limit for the crustal contribution to anisotropy in the Ryukyu area in Supplementary Figs S2 and S3.

We use the average shear wave splitting in the comparison of model results and the observations. For all observations used in this study, we also calculated the 95% confidence limits for the average shear wave splitting parameters *dt* (delay time) and *φ* (fast direction) by comparing the data to curves of the form *Si* = *dt sin*(2(*θ-φ*)). We used a *χ*^2^ analysis to determine appropriate CL ranges. The values of *χ*^2^ is defined as 
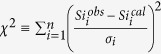
 where *i* is the number of the observed ray path, *Si*^*obs*^ and *Si*^*cal*^ are observed and calculated *Si* values, respectively, and *σ*_*i*_ is the error of *Si*^*obs*^ (Supplementary S1). The CL for the parameters *dt* and *φ* for the above form can be determined by calculating the value of Δ*x*^2^ for 2 degrees of freedom^39^. We use these 95% CL ranges for *dt* and *φ* as measurement errors in Figs 4, 5, 6 and Supplementary Figs S2 and S3.

We also incorporate uncertainties from errors in delay time estimates, the range of variation in the strength of CPO patterns, different possible averaging assumptions for calculating elastic properties, and estimates of the crustal contribution to anisotropy. If we take into consideration all uncertainties due to seismic wave observations, the crustal anisotropy, antigorite CPO patterns and the averaging scheme for CPO, data for the teleseismic events gives an estimate of ≥54% for the percentage of serpentinite present in the Ryukyu forearc mantle arc (Supplementary Fig. S2). In addition, we adopt the values for the proportion of antigorite and the error estimates derived from the analysis of teleseismic phases, and search for the distribution of antigorite within the wedge that best accounts for the observation of local-S phases. If we take into consideration all uncertainties, the minimum estimates for the distribution of serpentinite becomes ~66 km from the tip of the wedge (Supplementary Fig. S3). Based on these models, we estimate that the full range of antigorite domains with vertical and slab parallel foliations compatible with the observed wave splitting is ~66–100 km from the tip of the wedge. Our estimate for the degree of serpentinization would result in a bulk density similar to lower crustal material, which is compatible with the reported regional gravity low in the area^40^ (a more detailed comparison with other geophysical data is presented in the Supplementary Discussion).

The orientation of the antigorite foliation in the serpentinite is required to vary throughout the mantle wedge. The subduction boundary is a domain of strong shear and we, therefore, place a domain of foliated antigorite parallel to this boundary. The location of the steeply dipping domain is not well constrained, but our models and previous work^9^,^17^,^21^,^29^ propose the most reasonable pattern is a change from slab-parallel close to the slab through vertical in the middle of the wedge to horizontal in the shallowest part of the wedge.

### Geodynamic modeling of the Ryukyu arc

**Figure 7:**
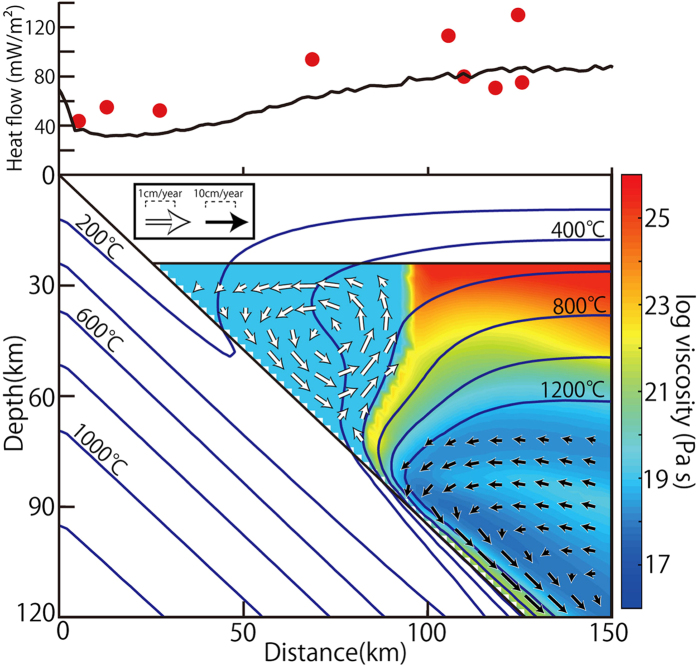
Dynamically calculated wedge mantle flow in the Ryukyu arc. Comparison of the predicted surface heat flow (thick line) with the observed values (red circles)^42^ is shown in the upper part. In the lower part, white and black arrows represent the calculated forearc mantle flow of the foliated antigorite-rich serpentinite domain and the induced flow of the olivine-rich mantle domain, respectively. The lengths of the arrows indicate the corresponding flow velocities. The antigorite domain flows more slowly than the olivine rich domain. Viscosity is indicated by the color shading and isotherms are indicated by blue lines drawn for 200 °C intervals.

The changes in foliation orientation derived from our analyses suggest that antigorite-bearing forearc mantle flows in the whole forearc region. To confirm that the flow pattern implied by our seismic studies is compatible with the geodynamic setting of the Ryukyu arc, we carried out numerical simulations of flow in the upper mantle wedge using parameters appropriate for this region including our results for the proportion and distribution of antigorite-rich serpentinite. In our model the forearc mantle where the temperature is less than 650 °C and the distance from the tip of the wedge is less than ~70 km consists of antigorite-rich serpentinite that has an effective viscosity of 10^19^ Pa s and reference density of 2.70 g cm^−3^ (equivalent to ~90% serpentinized peridotite based on densities of 2.62 g cm^−3^ for antigorite^9^). Our models are conservative in the sense that there is no coupling between the serpentinised domain and the downing slab. The results show that an anticlockwise convection forms in the serpentinized shallow wedge (Fig. 7). The pattern of convective mantle flow is in agreement with the orientation of antigorite constrained by our seismic anisotropy modeling (Figs 6a,b and 7). This result is compatible with previous numerical modeling that shows convective flow can be induced in the forearc wedge mantle when the viscosity of the wedge mantle is sufficiently low (<~4 × 10^19^ Pa s)^41^; compatible with estimates for the effective viscosity of antigorite^5^. In our model the dominant driving force for the convection in the shallow wedge is due to the horizontal density contrast associated with the dominantly horizontal thermal gradient and is not dependent on mechanical coupling between the downgoing slab and overlying shallow wedge. Introducing mechanical coupling between the downgoing slab and overlying serpentinized mantle would increase the rate of flow (see Table 1 regarding detail physical parameters of calculation). The flow in the serpentinized shallow mantle shown by our model is essentially independent from the deeper olivine-rich and higher temperature mantle (Fig. 7). The isolation of the shallow serpentinized mantle means that it will be associated with low surface heat flow compatible with observations of forearc regions^42^.

## Discussion

We suggest that subduction zones that exhibit a similar pattern of both local-S and SK(K)S observations as found in the Ryukyu arc are likely to have widespread antigorite-dominant shallow mantle that is undergoing convection. Examples where teleseismic SK(K)S events associated with trench-parallel fast directions show delay times that are equal to or larger than those of local-S phases (≥~1 s) with trench-parallel fast directions, include the Aleutians, Izu-Bonin, Tonga-Kermadec subduction zones^43^. This group of subduction zones includes a wide variety of slab ages implying that water transport to the shallow wedge mantle can occur irrespective of the age and thermal structure and suggests the general model for forearc mantle hydration being closely controlled by the age of the slab e.g.^10^ needs to be re-considered.

In this scenario, the reason why other subduction zones do not show large S-phase splitting is not fully determined, but a combination of slab geometry, the balance of the supply of water and dry mantle material into the wedge, and biases of source-receiver geometries are important topics for consideration. We note that subduction zones with large splitting times^43^ show relatively steep slab dips (~40–60° 42) and suggest that significant dip is required to allow room for convection with the formation of both slab parallel and sub-vertical foliation domains. The fact that forearc mantle associated with a shallow slab dip is never associated with large S-wave splitting is an important observation that supports our model.

## Methods

### Geometry of Ryukyu arc and approach to programming used in this modeling

For our model calculations of S-wave splitting, we used an approach that combines two recently developed Matlab toolkits: MTEX^44^,^45^ and MSAT^46^. This combination allows us to model seismic waves propagating with different ray paths through different domains of mechanically anisotropic rocks where the anisotropy is calculated from given CPO patterns of specified minerals. The natural antigorite CPO used in our calculations originated in the wedge mantle in an ancient subduction zone^31^. The calculated Voigt–Reuss–Hill average elasticity matrix of the antigorite aggregate is:
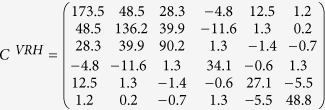
We traced rays in three dimensions through a model domain with seismic anisotropy defined on a grid with 2 km spacing using a straight-ray approximation derived from refs 29,30,32, and calculated shear wave splitting for each 1 km step-size in the ray. The similar large trench-parallel fast Vs associated with both teleseismic and intra slab events observed in Ryukyu implies that the anisotropies of the slab and the sub-slab in the deep upper mantle contribute little to the observed teleseismic S-wave splitting. Therefore in our model we follow earlier workers^29^,^30^ in assuming the teleseismic S-wave splitting is all due to anisotropy above the slab. In addition we assume the mantle of the Ryukyu arc region consists dominantly of olivine and antigorite. Studies of natural examples of deformed antigorite-bearing peridotites show olivine has only a very weak CPO probably as a result of slip along olivine-antigorite grain boundaries and rigid body rotation^47^. In addition the elastic anisotropy of antigorite is many times greater than that of olivine. The expected weak CPO of olivine and the strong anisotropy of antigorite mean that for our purposes we can consider the anisotropy of hydrated mantle in the Ryukyu arc to be solely due to the presence of antigorite. This conclusion is the same as other studies in the area (e.g. ref. 8). In Figs 4, 5, 6 we treat the colorless olivine-rich domains in the wedge mantle as seismically isotropic domains. In addition, in the comparison of the calculations with the observations for teleseismic SK(K)S phases, we used the limited ray paths from the observed ray paths reported by refs 29,30,32 so that selected ray paths encompass a range of backazimuths and incidence angles at each station. These selected ray paths are representative of the types of observed rays in the entire wedge mantle and have incident angles at the surface less than 40 degrees (required for reliable shear-wave splitting measurement). In the comparison with local-S phases, we used all observed ray paths that have low incidence angles (less than 40 degrees) selected from the paths reported in refs 29,30,32 (Fig. 3 and Supplementary Fig. S1). The aggregate splitting for each ray-path was calculated using a 4-layer model where the splitting operator for each domain was calculated using the Christoffel equations and these operators were combined using the multilayer splitting equations given in ref. 48 (see ref. 46 for further details of calculation). To perform the calculation, boundaries of different domains with different anisotropies need to be defined. The most important boundaries in our study are the subduction boundary along the upper surface of the slab and the base of the continental crust. Previous studies have shown that although some variability in slab geometry is present, the average slab dip is constant to within 10° and the trench geometry is approximately linear^29^. These observations mean the subduction geometry in the Ryukyu subduction zone can be closely approximated by a 2-D model^29^. We take a constant value for the slab dip of 43.5°^29^ and the trench axis of the Ryukyu subduction system, illustrated in Fig. 3, is rotated 33 degrees clockwise from north. Use of the dataset for the high-frequency bandpass filter leads to different resulting splitting parameters from that of low-frequency bandpass filter with the boundary placed around 0.125 Hz^49^. Here we used the dataset of observed low-frequency rays (0.02–0.125 Hz) for comparison with model calculations because both local-S and teleseismic phases with low-frequencies have been observed^30^,^32^. In our calculations we assumed a constant frequency of 0.10 Hz. The initial polarization angles of the events also affect the results of calculated delay time and fast direction^46^. For teleseismic SK(K)S phases we assume an initial polarization of the backazimuth to the event. For local events we calculate an initial polarization based on best fitting double-couple earthquake mechanism (strike, dip and slip) that are collected by the Japanese *F*-net array and calculated from CMT solution for each event. We used the seismic data sources observed in the broadband seismograph network “*F*-net” that are available from the website of Japanese National Research Institute for Earth Science and Disaster Prevention (http://www.fnet.bosai.go.jp/event/search.php?LANG=en).

### Geodynamic calculation of flow in the wedge mantle

The geodynamic modeling used to simulate flow in the wedge mantle of the Ryukyu arc uses a 200 km deep × 316 km wide model domain with 100 × 150 grids and ca 250,000 marker that covers the region from the trench to back-arc. For the slab dip and the thickness of the continental crust, we use values appropriate for the Ryukyu arc of 43.5° and 24 km, respectively^29^. The present 2-D model consists of a kinematically prescribed subducting slab (including 7 km thick oceanic crust), a viscous mantle wedge in which the flow field is to be calculated, and an overriding rigid 24 km thick continental crust (12 km upper crust and 12 km lower crust). The mantle wedge consists of serpentinized peridotite with constant viscosity (10^19^ Pa s) and olivine-rich peridotite that flows by dislocation creep^50^ assuming constant water content in olivine (1000 ppm H/Si). The governing equations for the flow and temperature fields are conservation equations of mass, momentum, and energy, with the Boussinesq approximation. The code used in this study for the calculation is based on the published numerical code I2VIS^51^ that uses finite differences with a marker-in-cell technique. We use the velocity boundary condition along the slab-wedge interface: no coupling (v = 0 cm yr^−1^) for the depth range 0–80 km and complete coupling (v = 6.95 cm yr^−1^) at depths > 90 km with a transitional zone (80–90 km). The velocities given by an analytical solution for the corner flow^52^ are assigned to the boundary conditions for the right vertical boundary and the horizontal bottom boundary of mantle wedge. The top boundary of the mantle wedge is rigid (v = 0 cm yr^−1^). The continental geotherm, assigned to both the initial thermal condition and the back-arc side boundary condition, is based on a 1D steady-state conduction model that incorporates radiogenic heat production that gives a surface heat flow of 75 mW m^−2^. The deeper part (>67 km) is assigned a geotherm calculated assuming a mantle potential temperature of 1350 °C and an adiabatic temperature gradient of 0.3 °C km^−1^. The oceanic geotherm follows the plate cooling model^53^,^54^, assuming a plate thickness of 106 km and mantle potential temperature of 1390 °C^55^. The temperature of oceanic asthenosphere (depth deeper than 106 km) are calculated assuming an adiabatic temperature gradient of 0.3 °C km^−1^. The age of the incoming slab is taken to be 43 Ma^56^. The thermal boundary condition for the left and right inflow boundary are fixed thermal structures, that is the oceanic geotherm taking the subduction angle into account and the continental geotherm, respectively. The bottom outflow boundary is adiabatic and the top surface has a constant temperature T = 0 °C. Frictional heating along the decoupled slab surface is incorporated using a frictional coefficient of 0.01 up to a depth of 48 km, which reduces linearly to 0 at a depth of 80 km. The energy equation also includes radioactive, adiabatic and viscous heating^51^. The physical parameters used in the calculation are listed in Table 1. Figure 7 shows the result after 20 My of subduction.

**Code availability.** The code used to generate the predictions of seismic anisotropy can be accessed at https://github.com/jwookey/SiMMS. The original code I2VIS used to generate the predictions of the wedge mantle flow is provided in ref. 51.

## Additional Information

**How to cite this article**: Nagaya, T. *et al*. Seismic evidence for flow in the hydrated mantle wedge of the Ryukyu subduction zone. *Sci. Rep.* **6**, 29981; doi: 10.1038/srep29981 (2016).

## Supplementary Material

Supplementary Information

## Figures and Tables

**Figure 1:**
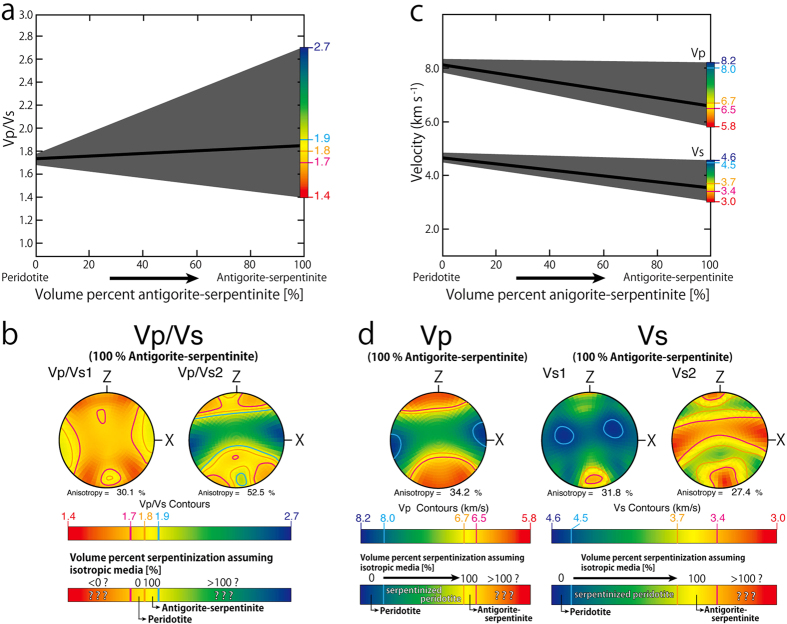
The range of possible Vp/Vs values and seismic velocities (Vp and Vs) for different proportions of serpentinization taking into account the anisotropy. (**a**) Relationship between the proportion of serpentinization and Vp/Vs. The thick black line represents Vp/Vs ratio based on average seismic velocities of peridotite and antigorite-serpentinite^57^. The gray region shows the possible range taking into account the anisotropies of antigorite^31^ and olivine^57^. The color bar is the same as for (**b**). (**b**) Anisotropy in the Vp/Vs ratio of the antigorite-serpentinite. Plots are all lower-hemisphere equal-area. Foliation and lineation parallel to X-axis and X-Y plane, respectively. The contours show the same values in the color bar. Prepared using software of D. Mainprice^58^ and antigorite CPO from ref. 31. (**c**) The relationship between the proportion of antigorite in serpentinized peridotite and seismic values (Vp and Vs). The anisotropies of antigorite-serpentinite and peridotite are taken from refs 31,57, respectively. The color bar is the same as for (**d**). Other features are the same as in (**a**). (**d**) Anisotropic seismic properties (Vp and Vs values) correspond to the fabric of the antigorite-serpentinite of ref. 31. Other features are the same as in (**b**).

**Figure 2:**
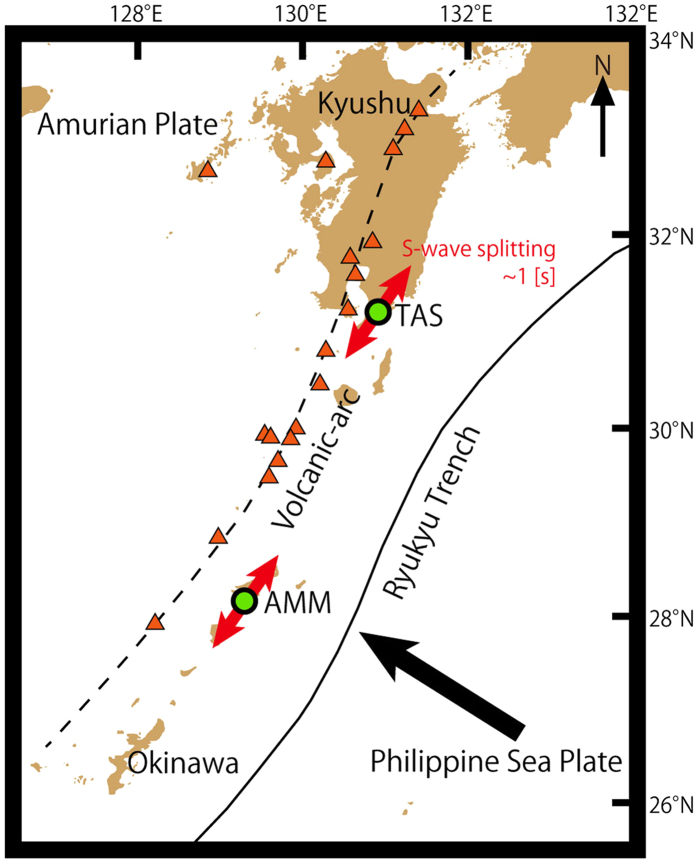
Observed S-wave splitting and ray paths in the Ryukyu subduction system. Geometry and seismic anisotropy of Ryukyu subduction system based on ref. 29. Green circles and red triangles show the locations of the seismic stations AMM and TAS and volcanic centers respectively. The large black arrow denotes plate motion of the Philippine Sea Plate with respect to the Amurian Plate. Large time delays between fast and slow S-waves (delay time) were observed at AMM and TAS^30^,^32^. The map was created using Adobe Illustrator CS5 software.

**Table 1:** The physical parameters used for the mantle flow calculation.

	Olivine-rich peridotite	Serpentinized peridotite	Oceanic crust	Lower continental crust	Upper continental crust
Reference density at 0 °C and 0.1 Pa (kg m^−3^)	3300	2700	3100	3100	2700
Radioactive heat production (W m^−3^)	0	0	2.7 × 10^−7^	2.7 × 10^−7^	1.3 × 10^−6^
Thermal diffusivity (mm^2^ s^−1^)*	Value for Fo_90_Fa_10_**		Value for crust		
Thermal expansion (K^−1^)	3.0 × 10^−5^				
Compressibility (GPa^−1^)	1.0 × 10^−11^				
Specific heat (J kg^−1^ K^−1^)	1200				

^*^The general form and mineral physics coefficients for thermal diffusivity are derived from ref. 60.

^**^Fo = Forsterite, Fa = Fayalite.
