# Supplementary material for: Seismic evidence for flow in the hydrated mantle wedge of the Ryukyu subduction zone
Source: Sci Rep. 2016 Jul 20;6:29981. doi: 10.1038/srep29981 (PMC4951697; doi:10.1038/srep29981)
Supplement: Supplementary Information [file srep29981-s1.pdf]

Seismic evidence for flow in the hydrated mantle wedge of the Ryukyu subduction zone

Takayoshi Nagaya[1,2,3,*], Andrew M. Walker[2,4], James Wookey[2], Simon R. Wallis[1], Kazuhiko Ishii[5] & J.-Michael Kendall[2]

[1]Graduate School of Environmental Studies, Nagoya University, Nagoya 464-8601, Japan.

[2]School of Earth Sciences, University of Bristol, Bristol BS8 1RJ, UK. [3]Graduate School of Environmental Studies, Tohoku University, Sendai 980-8579, Japan. [4]School of Earth and Environment, University of Leeds, Leeds LS2 9JT, UK. [5]Graduate School of Science, Osaka Prefecture University, Sakai 599-8531, Japan.

[*]Current e-mail:t.nagaya@geo.kankyo.tohoku.ac.jp

Supplementary Information

This file presents additional discussion of the comparison with geophysical observations and 3 additional Figures.

Comparison with the geophysical observations

The above modeling based on the observed shear wave splitting in the Ryukyu arc gives estimates for the proportion and distribution of antigorite-rich domains of at least ~54% and at least ~66 km from the tip of the wedge. This degree of serpentinization in the Ryukyu arc should be associated with other geophysical characteristics. Gravity observations show a strong negative free air anomaly of ~−70–−100 mgal with a maximum magnitude of −130 mgal. This low-density domain of the fore-arc mantle has been identified from southern Kyushu[40,61] to Ryukyu[62,63] and may continue beyond this region. The low-density domain has been identified close to both the AMM and TAS stations. The location and values of the gravity low are compatible with our estimates for the degree of serpentinization and the predicted low fore-arc mantle density (2.62–2.94 g cm^{-3} based on densities of 2.62 g cm^{-3} for antigorite[9] and 3.32 g cm^{-3} for olivine[57]).

Receiver function (RF) analysis from the Ryukyu arc[64] suggests the existence of multiple anisotropic layers in the shallow wedge mantle including anisotropic layers with a

fast velocity direction parallel to the trench. The presence of domains of antigorite-rich foliated serpentinite in the wedge mantle at varying angles to the slab, as proposed in our model, results in a similar seismic velocity anisotropy. The earlier study (ref. 64) assumes that the mantle wedge above the serpentinized shear zone along the plate boundary is olivine-rich and capable of deforming by dislocation creep at high differential stress[e.g. 65–68]. However, geodynamic and petrological modelling suggests the high stresses and relatively high temperatures needed to maintain these conditions will not be present in the forearc of the Ryukyu arc or indeed most other convergent margins[42,47]. In addition, the anisotropy expected for olivine CPO patterns is insufficient to account for the large shear wave splitting[e.g. 8].

In addition, seismic tomography in the Ryukyu arc reveals the existence of domains of low Vp and Vs velocities and high Vp/Vs values[40,62,69]. Based on the experiments for the average seismic velocities, antigorite-bearing serpentinite shows Vp/Vs, Vp and Vs values of ~1.8–1.9, ~6.5–6.7 km/s and ~3.4–3.7 km/s, respectively[e.g. 14,15] and dry peridotite shows Vp/Vs, Vp and Vs values of ~1.7–1.8, ~8.0–8.6 km/s and ~4.5–4.9

km/s [e.g. 14,16], respectively. This means serpentinized peridotite shows intermediate values of Vp/Vs ratio, Vp and Vs between antigorite-serpentinite and peridotite. For aligned antigorite, the anisotropies of the Vp/Vs_1, Vp/Vs_2, Vp, Vs_1 and Vs_2 given as the values of 100(max − min)/[(max + min)/2] are 30.1%, 52.5%, 34.2%, 31.8% and 27.4%, respectively (Fig. 1 of main paper). Therefore when these anisotropies are taken into account, the Vp/Vs ratio can vary 1.4–2.7 and the values of Vp and Vs can vary 5.8–8.2 km/s and 3.0–4.6 km/s, respectively depending on the propagation path of the seismic wave (Fig. 1 of main paper). This wide range of possible values of Vp/Vs, Vp and Vs covers the full range of possible values for mantle and clearly shows that the proportion of serpentinization of mantle peridotite cannot be unambiguously determined from only the Vp, Vs and Vp/Vs observations in the subduction zones—some consideration of anisotropy is also needed. Nevertheless, a range of relatively high Vp/Vs ratios and low seismic velocities (Vp and Vs) can be identified which is only compatible with the presence of serpentinite-rich domains. The seismic velocity observations in southern Kyushu[40] and

Ryukyu[62,69] fall within the range indicating the existence of serpentinite and support our proposed existence of a serpentinite-rich domain in the forearc mantle.

In summary, our estimate for the proportion and distribution of an antigorite-rich domain in the Ryukyu forearc mantle is in good agreement with the results of gravity data, RF analysis and seismic tomography. However, our new approach provides constraints on these features that far exceed the resolution provided by gravity observations, RF analysis or seismic velocity tomography.

Supplementary References

61. Nakada, M. et al. Late Pleistocene crustal uplift and gravity anomaly in the eastern part of Kyushu, Japan, and its geophysical implications. *Tectonophysics* **351**, 263–283 (2002).

62. Wang, Z., Huang, R., Huang, J. & He, Z. P-wave velocity and gradient images beneath the Okinawa Trough. *Tectonophysics* **455**, 1–13 (2008).

63. Lin, J.-Y., Sibuet, J.-C., Hsu, S.-K. & Wu, W.-N. Could a Sumatra-like megathrust earthquake occur in the south Ryukyu subduction zone? *Earth Planet. Space* **66**, 49, doi:10.1186/1880-5981-66-49 (2014).

64. McCormack, K., Wirth, E.A. & Long, M.D. B-type olivine fabric and mantle wedge serpentinization beneath the Ryukyu arc. *Geophys. Res. Lett.* **40**, 1–6 (2013).

65. Jung, H. & Karato, S. Water-induced fabric transitions in olivine. *Science* **293**, 1460–1463 (2001).

66. Kneller, E.A., van Keken, P.E., Karato, S. & Park, J. B-type olivine fabric in the mantle

wedge: Insights from high-resolution non-Newtonian subduction zone models. *Earth Planet. Sci. Lett.* **237**, 781–976 (2005).

67. Jung, H., Katayama, I., Jiang, Z., Hiraga, T. & Karato, S. Effect of water and stress on the lattice-preferred orientation of olivine. *Tectonophysics* **421**, 1–22 (2006).

68. Katayama, I. & Karato, S. Effect of temperature on the B- to C-type olivine fabric transition and implication for flow pattern in subduction zones. *Phys. Earth Planet. Inter.* **157**, 33–45 (2006).

69. Nakamura, M., Yoshida, Y., Zhao, D., Katao, H. & Nishimura, S. Three-dimensional P- and S-wave velocity structures beneath the Ryukyu arc. *Tectonophysics* **369**, 121–143 (2003).

Supplementary Figures and Figure legends

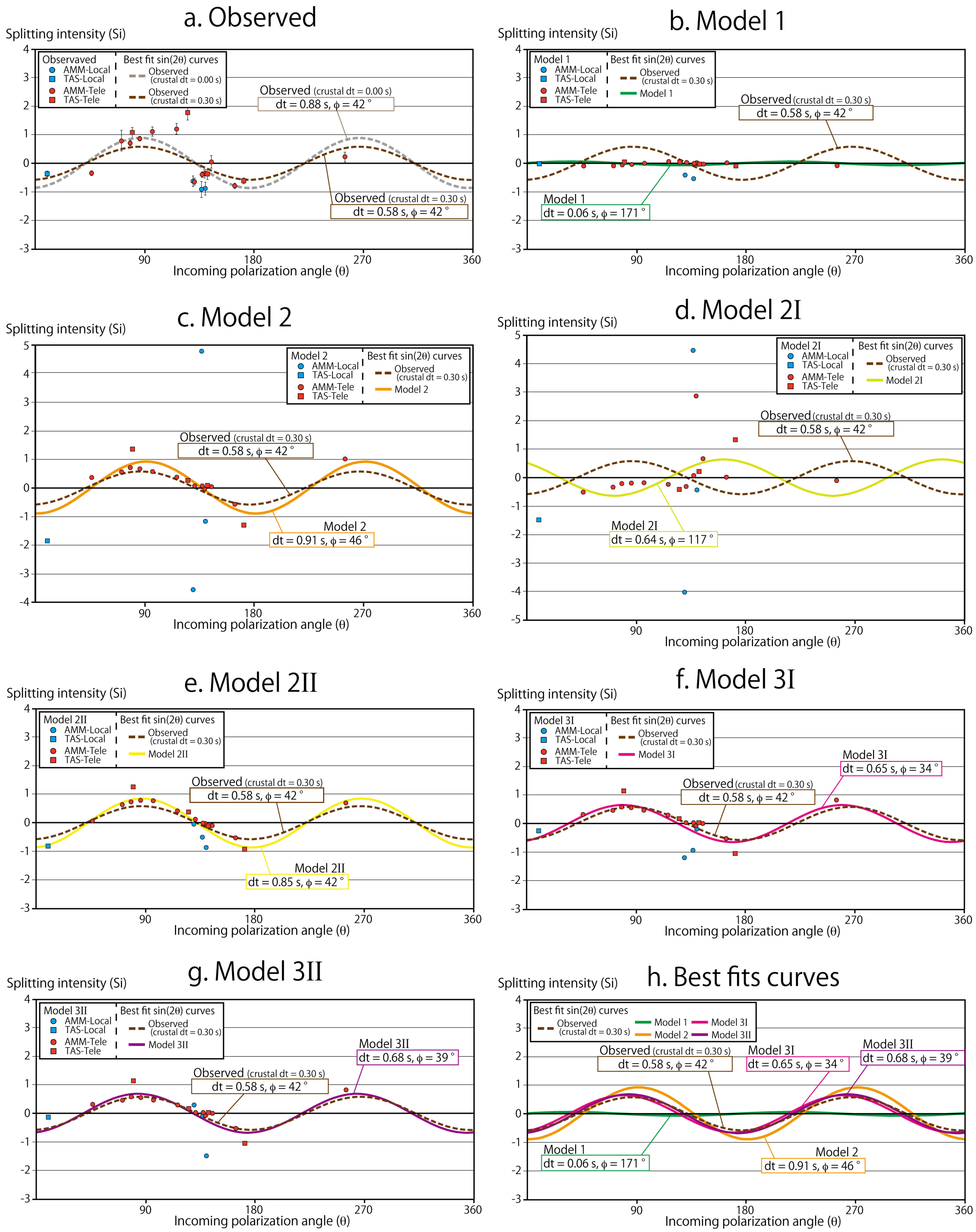
a. Observed
Splitting intensity (Si)
Observaved
AMM-Local
TAS-Local
AMM-Tele
TAS-Tele
Best fit sin(2θ) curves
Observed (crustal dt = 0.00 s)
Observed (crustal dt = 0.30 s)
Observed (crustal dt = 0.00 s)
dt = 0.88 s, φ = 42 °
Observed (crustal dt = 0.30 s)
dt = 0.58 s, φ = 42 °
Incoming polarization angle (θ)
b. Model 1
Model 1
Observed (crustal dt = 0.30 s)
dt = 0.58 s, φ = 42 °
Model 1
dt = 0.06 s, φ = 171 °
c. Model 2
Model 2
dt = 0.91 s, φ = 46 °
d. Model 2I
Model 2I
dt = 0.64 s, φ = 117 °
e. Model 2II
Model 2II
dt = 0.85 s, φ = 42 °
f. Model 3I
Model 3I
dt = 0.65 s, φ = 34 °
g. Model 3II
Model 3II
dt = 0.68 s, φ = 39 °
h. Best fits curves
Best fit sin(2θ) curves
Observed (crustal dt = 0.30 s)
Model 1
Model 2
Model 3I
Model 3II

Supplementary Figure S1 The average delay time and fast direction using incoming polarization angle and splitting intensity. Tele- (red) and local- (blue) phases are shown for the observations with 2-σ error bars[29,30,32] and the calculation in each model. The different sites are distinguished by circles (AMM) and squares (TAS). The average for both phases are determined by the best-fit sin (2θ) curve. **a**, The dotted lines indicate the curve for the observations (gray) and it with the maximum crustal anisotropy[35,36] (brown). **b**, **c, d, e, f, g,** Model 1 (b), 2 (c), 2I (d), 2II (e), 3I (f), 3II (g). The solid line indicates the curve for the calculation. **h**, Compilation of the observation and models. This calculation includes an assumed crustal anisotropy of 0.3 s with trench-parallel splitting[35,36].

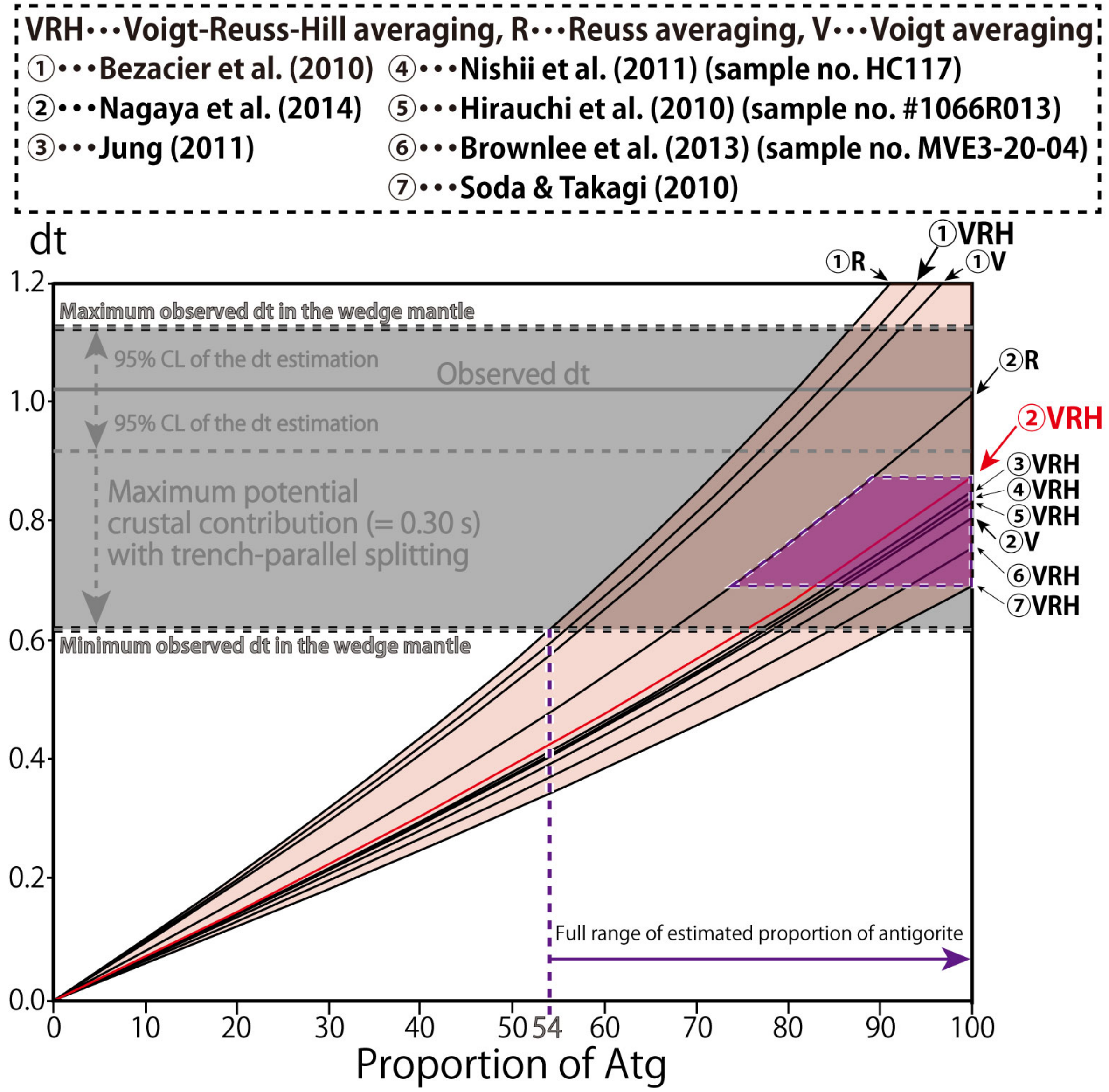

Supplementary Figure S2 The proportion of antigorite in the model 3I and predicted average delay time in the wedge mantle for teleseismic phases. Red line denotes the average delay time in the wedge mantle predicted in model 3I using teleseismic phases. The light pink domain shows the effect of applying different averaging schemes and use of

different CPO patterns for strongly foliated natural examples of antigorite-serpentinite (see figure for references). The gray domain denotes the range in possible delay times in the wedge mantle including 95% CL for teleseismic events and uncertainties in crustal anisotropy[35,36]. The purple area represents our best estimate for the ranges of mantle wedge delay times, proportion of antigorite and strength of the corresponding CPO.

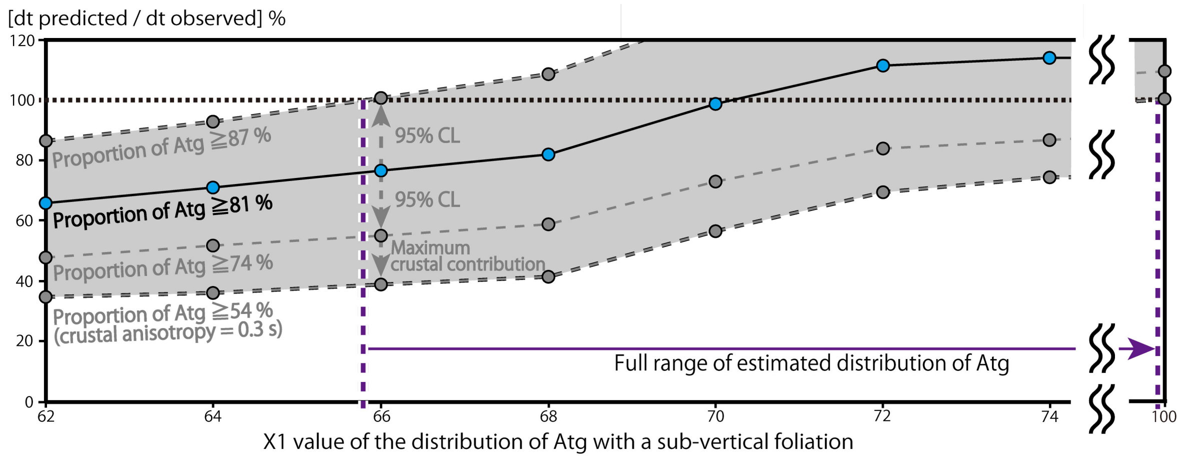

Supplementary Figure S3 Comparison between the observed delay times for local-S phases and those calculated using model 3I for a varying lateral extent of the sub-vertical antigorite domain. The black line connecting blue circles denotes the ratio of the average predicted delay time to the average observed delay time in the wedge mantle for local-S phases. The gray range denotes the full range including 95% CL for the delay time estimates and a maximum estimate for the crustal anisotropy. Values for the proportion of antigorite are determined by the teleseismic modeling results shown in Supplementary Fig. S2.
